# ecg2o: a seamless extension of g2o for equality-constrained factor graph optimization

**DOI:** 10.3389/frobt.2025.1698333

**Published:** 2026-01-20

**Authors:** Anas Abdelkarim, Daniel Görges, Holger Voos

**Affiliations:** 1 Interdisciplinary Centre for Security, Reliability and Trust (SnT), University of Luxembourg, Luxembourg, Luxembourg; 2 Department of Electrical and Computer Engineering (EIT), RPTU University of Kaiserslautern-Landau, Kaiserslautern, Germany

**Keywords:** constrained factor graphs, optimal control, SLAM, equality-constrained optimization, SQP

## Abstract

Factor graph optimization serves as a fundamental framework for robotic perception, enabling applications such as pose estimation, simultaneous localization and mapping (SLAM), structure-from-motion (SfM), and situational modeling. Traditionally, these methods solve unconstrained least squares problems using algorithms such as Gauss-Newton and Levenberg-Marquardt. However, extending factor graphs with native support for hard equality constraints can yield more accurate state estimates and broaden their applicability, particularly in planning and control. Prior work has addressed equality handling either by soft penalties (large weights) or by nested-loop Augmented Lagrangian (AL) schemes. In this paper, we propose a novel extension of factor graphs that seamlessly incorporates hard equality constraints without requiring additional optimization techniques. Our approach maintains the efficiency and flexibility of existing second-order optimization techniques while ensuring constraint satisfaction. To validate the proposed method, an autonomous-vehicle velocity-tracking optimal control problem is solved and benchmarked against an AL baseline, both implemented in g2o. Additional comparisons are conducted in GTSAM, where the penalty method and AL are evaluated against our g2o implementations. Moreover, we introduce ecg2o, a header-only C++ library that extends the widely used g2o library with full support for hard equality-constrained optimization. This library, along with demonstrative examples and the optimal control problem, is available as open source at https://github.com/snt-arg/ecg2o.

## 1 Introduction

Factor graphs are extensively used in robotic perception tasks for efficiently modeling large-scale probabilistic inference problems (Dellaert and Kaess, 2017). These methods use graph-based optimization techniques, such as weighted least squares, to address key problems like SLAM and situational awareness (Tourani et al., 2022; Bavle et al., 2022). Typically, these optimization problems are addressed using second-order unconstrained algorithms, including Gauss-Newton (Lai et al., 2017) and Levenberg–Marquardt (Moré, 2006). Several well-established libraries, such as GTSAM (Dellaert et al., 2022), g2o (Kümmerle et al., 2011), and SRRG2 (Grisetti et al., 2020), have been developed to provide robust back-end implementations of these optimization algorithms, along with user-friendly front-end interfaces tailored for robotic applications.

Recent advancements in the literature have extended factor graph–based optimization beyond perception tasks to also encompass optimal control applications. The primary challenge in this extension lies in handling constraints, where optimal control problems inherently involve hard constraints, unlike perception problems which are typically soft-constrained. However, factor graph optimization can provide a unified framework for perception and control tasks in robotics. Furthermore, this approach facilitates the reuse of established and computationally efficient algorithms available in factor graph libraries (Abdelkarim et al., 2025). In contrast, traditional optimization frameworks widely used in optimal control, such as CasADi (Andersson et al., 2019), AMPL (Abdelkarim and Zhang, 2020; Abdelkarim et al., 2023), and IPOPT (Wächter and Biegler, 2006), although highly effective, do not naturally integrate with factor graph–based optimization frameworks. This disconnect can introduce computational inefficiencies and added complexity, particularly in robotic applications that demand a seamless integration of perception and control.

It is important to highlight that in optimal control, hard equality constraints define strict and deterministic relationships between variables. A typical example is the system dynamics, which must be satisfied exactly to ensure that state transitions remain physically consistent. In contrast, the motion and sensor models in SLAM establish probabilistic or soft constraints between variables. These constraints do not enforce exact relationships but instead incorporate uncertainty directly into the factor graph formulation. As a result, hard equality constraints enforce exact dependencies to guarantee consistency, e.g., with the underlying physics, while soft probabilistic constraints model uncertainty and enable more flexible estimation.

In this work, we address the integration of hard equality constraints within factor graphs. Our contributions can be summarized as follows: (i) Method: we introduce an Sequential Quadratic Programming (SQP)-inspired approach that leverages the Karush-Kuhn-Tucker (KKT) conditions to natively integrate hard equality constraints into the factor graph framework. (ii) Implementation: we provide a lightweight, header-only C++ library, ecg2o, that extends the g2o optimization framework with support for both the Augmented Lagrangian (AL) method and the proposed KKT-based approach. The library is publicly available at https://github.com/snt-arg/ecg2o to foster reproducibility and further research. In addition, we introduce a stopping criterion based on the norm of the update step in g2o, ensuring termination once the step size becomes sufficiently small. (iii) Validation: we conduct a case study on trajectory-tracking optimal control, demonstrating the effectiveness of the proposed method. Furthermore, we benchmark our implementations against the penalty and AL methods in GTSAM, highlighting the efficiency and robustness of the implemented approaches.

The remainder of this paper is organized as follows. Section 2 reviews the state of the art in factor-graph-based optimization and motivates the need for more efficient methods for equality constraint handling. Section 3 introduces the necessary preliminaries, including weighted least squares, Gauss–Newton for unconstrained optimization, and the soft-constraint formulation. Section 4 presents the AL and the proposed KKT-based Gauss–Newton approaches. Section 5 reports the experimental evaluation comprising two case studies: trajectory-tracking optimal control and a nonlinear least-squares problem with equality constraints, where the proposed implementation is also compared against the penalty and AL methods in GTSAM. Finally, Section 6 concludes the paper and outlines directions for future work.

## 2 State of the art and motivation

### 2.1 State of the art

While factor graphs have initially been introduced to handle robotic perception tasks, they are increasingly used also for robotic optimal control problems, as summarized in the comprehensive review (Abdelkarim et al., 2025) along with a description of various methods for contsraint handling. In the following, we provide an overview of the key literature in this area.

Chen et al., 2019 introduced a general framework for applying Linear Quadratic Regulators (LQR) using factor graphs. In this framework, equality constraints are used to enforce system dynamics and are incorporated into the cost function as a weighted least squares term. By assigning a sufficiently large weighting matrix, these equality constraints effectively act as soft constraints, which are approximately satisfied as the weighting approaches infinity.

Yang et al. (2021) extended this approach by introducing additional equality constraints between variables within the LQR framework. Factor graph–based LQR has been applied to a variety of domains, including wireless mesh network control (Darnley, 2021), tactile estimation and extrinsic contact control (Kim et al., 2023), and trajectory generation for graffiti robots (Chen et al., 2022a; Chen et al., 2022b). Notably, these implementations have primarily relied on the GTSAM library to formulate and solve the optimization problems.

Beyond LQR-based approaches, more advanced constrained optimization techniques have been explored for handling equality constraints, most notably the AL method (Bertsekas, 2014). The fundamental idea of AL is to introduce Lagrange multiplier terms and a quadratic penalty term for constraint violations into the cost function, thereby forming the AL function. The optimization process involves two nested loops: the inner loop minimizes the AL function while keeping the Lagrange multipliers fixed, while the outer loop updates the Lagrange multipliers and penalty parameters until a specified stopping criterion is met.

The AL method has been implemented in GTSAM for improved state estimation (Sodhi et al., 2020) and has been applied in navigation and 3D manipulation planning (Qadri et al., 2022). Furthermore, the AL has been utilized in SRRG2 for localization and control of unicycle robots (Bazzana et al., 2022) and for pseudo-omnidirectional platform control (Bazzana et al., 2024).

While these methods have been successfully applied to various robotics and control problems, existing approaches still suffer from key limitations as described below.

### 2.2 Motivation

Incorporating equality constraints into factor graphs has been primarily achieved through two approaches: soft constraints and the AL method. In the soft constraints approach, enforcing equality constraints requires assigning an infinitely large weighting matrix, which is not feasible in practical implementations. Instead, a large but finite weighting matrix is typically used. However, choosing an appropriate weight requires careful tuning to achieve balance between effectively enforcing the constraint and avoiding ill-conditioned optimization problems. If the original optimization problem is already ill-conditioned, determining an appropriate weighting matrix becomes even more challenging Abdelkarim et al. (2025).

In contrast, the AL method systematically enforces equality constraints without requiring direct weight tuning. However, the AL has notable limitations. First, as previously mentioned, it employs a nested loop structure, which may lead to unnecessary iterations in the inner loop. Second, the performance of the AL method is highly dependent on hyperparameter tuning Nocedal and Wright (2006), including the initial penalty term, penalty update factor, maximum penalty value, inner-loop iteration limit, and stopping criteria. Suboptimal parameter selection can significantly degrade performance.

To address these limitations, we propose an alternative approach that extends the Gauss-Newton and Levenberg–Marquardt methods, which are the primary unconstrained optimization algorithms used in factor graphs, by incorporating KKT conditions to explicitly enforce equality constraints. This approach offers faster convergence and eliminates the need for extensive hyperparameter tuning. Furthermore, despite g2o′s efficiency in robotic perception tasks, it does not provide built-in mechanisms for explicitly handling equality constraints. This limitation necessitates either manual constraint encoding using soft constraints (which leads to tuning challenges) or extending the solver framework itself.

## 3 Preliminaries

### 3.1 Weighted least squares in factor graphs

Factor graphs consist of variable nodes and factor nodes and represent a factorization of probability density functions (PDFs), which are typically assumed to follow a Gaussian distribution. In maximum *a posteriori* (MAP) inference, the objective is to maximize the factorized probability function, mathematically formulated in Equation 1.

$$X^{\text{MAP}}=\underset{X}{\text{argmax}}\overset{r}{\underset{j=1}{\prod }}\exp\left(-\frac{1}{2}||e_{j}\left(X_{j}\right)|{|}_{\Omega_{j}}^{2}\right).$$ (1)

Here, exp() represents the exponential function, $||e|{|}_{\Omega }^{2}=e^{\text{T}}\Omega e$ denotes the Mahalanobis norm, and $.^{\text{T}}$ is the transpose operator for vectors or matrices. The term $e_{j}$ is an error function associated with the factor j , which depends on a subset of the variable nodes, and $\Omega_{j}$ is the information matrix of factor j , computed as the inverse of the covariance matrix. Furthermore, X is the vector containing all variable nodes $X_{j}$ .

Since maximizing the MAP objective is equivalent to minimizing the negative log-likelihood, this optimization problem can be reformulated as a weighted least squares problem (Abdelkarim et al., 2025, § III.B)

$$X^{\text{MAP}}=\underset{X}{\mathrm{arg min}}\;\overset{r}{\underset{j=1}{\sum }}||e_{j}\left(X_{j}\right)|{|}_{\Omega_{j}}^{2}.$$ (2)

In the following sections, we present solutions to the optimization problem defined in Equation 2, first without hard constraints and then with incorporated hard equality constraints.

### 3.2 Gauss-newton method for unconstrained least-squares

In factor graphs, the factor nodes typically define a nonlinear error function. Thus, optimization methods operate on a linearized version of this function. This is achieved through first-order Taylor expansion described in Equation 3.

$$e_{j}\left(\underset{X_{j}}{\underbrace{\tilde{X_{j}}+\Delta X_{j}}}\right)\approx {\hat{e}}_{j}\left(\Delta X_{j}\right)=e_{j}\left(\tilde{X_{j}}\right)+J_{e_{j}}\left(\tilde{X_{j}}\right)\Delta X_{j},$$ (3)

where $\tilde{X_{j}}$ is a linearization point and $Je_{j}\left(\tilde{X_{j}}\right)$ is the Jacobian matrix of the error function of the factor j evaluated at the linearization point $\tilde{X_{j}}$ .

Applying the stationarity condition (the gradient of the cost function vanishes at an optimal point), the Gauss-Newton update step at iteration i is computed as

$$\underset{{\text{H}}^{i}}{\underbrace{\overset{r}{\underset{j=1}{\sum }}\text{map}\left({\text{H}}_{j}^{i}\right)}}\Delta X_{gn}^{i}=\underset{b^{i}}{\underbrace{\overset{r}{\underset{j=1}{\sum }}\text{map}\left(b_{j}^{i}\right)}},$$ (4)

where the contribution of the factor j at iteration i in building the linear system is

$${\text{H}}_{j}^{i}=||J_{e_{j}}\left(X_{j}^{i}\right)|{|}_{\Omega_{j}}^{2}$$ (5a)

$$b_{j}^{i}=-{J_{e_{j}}\left(X_{j}^{i}\right)}^{\text{T}}\Omega_{j}e_{j}\left(X_{j}^{i}\right),$$ (5b)

and map (⋅) , in Equation 4, is an operator that maps the local ${\text{H}}_{j}^{i}$ and $b_{j}^{i}$ to the global ${\text{H}}^{i}$ and $b^{i}$ . This mapping accounts for the fact that error functions generally depend only on subsets of the variable nodes, and map (⋅) ensures proper zero-padding where necessary.

Constructing and solving this linear system, presented in Equation 5, is a key computational step in factor graph optimization. Once the linear system is solved, the variable nodes are updated in each iteration as described in Equation 6.

$$X^{i+1}=X^{i}+\Delta X_{gn}^{i}.$$ (6)

Although the linear system formulation remains the same across unconstrained optimization methods, different techniques introduce modifications. For example, in the Levenberg-Marquardt method, numerical stability is improved by adding a positive damping term to the diagonal elements of the matrix H , mitigating issues related to ill-conditioned matrices.

### 3.3 Equality constraints as soft constraints

For vector-valued equality constraints $h_{j}\left(X_{j}\right)=0$ , a common approach is to add a quadratic penalty $||h_{j}\left(X_{j}\right)|{|}_{W_{h_{j}}}^{2}$ with a large positive-definite (typically diagonal) weighting matrix $W_{h_{j}}$ to the factor-graph objective in (2). This yields the unconstrained problem as presented in Equation 7.

$$\underset{X}{\text{min }}\;\overset{r}{\underset{j=1}{\sum }}||e_{j}\left(X_{j}\right)|{|}_{\Omega_{j}}^{2}+\overset{l}{\underset{j=1}{\sum }}||h_{j}\left(X_{j}\right)|{|}_{W_{h_{j}}}^{2}.$$ (7)

In a factor graph, this is implemented by adding one cost factor per constraint with error $e_{h_{j}}=h_{j}\left(X_{j}\right)$ and information matrix $\Omega_{h_{j}}=W_{h_{j}}$ . In the limit $W_{h_{j}}\;\to \infty$ , the optimizer drives $h_{j}\left(X_{j}\right)\;\to \;0$ . In practice, however, very large weights can cause ill-conditioning and sensitivity to variable/constraint scaling, so careful (problem-dependent) weighting or normalization is required.

## 4 Methodology

We consider the optimization problem formulated in Equation 2, now extended with linearly independent equality constraints:

$$\underset{X}{\text{min }}\;\overset{r}{\underset{j=1}{\sum }}||e_{j}\left(X_{j}\right)|{|}_{\Omega_{j}}^{2}$$ (8a)

$$\text{s.t.}\;h_{j}\left(X_{j}\right)=0\;\;j=1,\ldots ,l$$ (8b)

The associated Lagrangian (Abdelkarim, 2020, § 2.4.2) for the optimization problem in Equation 8 is given in Equation 9.

$$L\left(X,\gamma \right)=\overset{r}{\underset{j=1}{\sum }}||e_{j}\left(X_{j}\right)|{|}_{\Omega_{j}}^{2}+\overset{l}{\underset{j=1}{\sum }}{\gamma_{h_{j}}}^{\text{T}}h_{j}\left(X_{j}\right),$$ (9)

where the first term is the standard factor-graph objective (regular factors) and the second term introduces the Lagrange multipliers $\gamma_{h_{n}}$ for the equality constraints.

This section provides a brief overview of the two methods implemented in ecg2o: the AL method and the KKT-based approach. Throughout, we use $||v|{|}_{W}^{2}=v^{\text{T}}Wv$ .

### 4.1 Augmented lagrangian method

Inspired by the approach presented in (Bazzana et al., 2024), we implemented the AL method in ecg2o to enforce equality constraints. Particularly, the AL method adds a penalty term $||h_{j}\left(X_{j}\right)|{|}_{{\text{P}}_{h_{n}}}^{2}$ for each equality constraint, to the Lagrangian function. This results in the following augmented Lagrangian function:

$$L_{\text{aug}}\left(X,\gamma \right)=L\left(X,\gamma \right)+\overset{l}{\underset{n=1}{\sum }}||h_{n}\left(X_{n}\right)|{|}_{{\text{P}}_{h_{n}}}^{2},$$ (10)

where the penalty term in Equation 10 is weighted by the matrix described in Equation 11.

$${\text{P}}_{h_{n}}=\text{diag}\left(\rho_{h_{n},1},\ldots ,\rho_{h_{n},d}\right)$$ (11)

and diag(⋅) represents a diagonal matrix of dimension d . The penalty parameter ρ . Can either be uniform across all constraints or vary based on the algorithm’s settings. Here, we assume a uniform ρ . For all constraints.

Notably, in the AL, the penalty terms do not need to be excessively large, as discussed in (Nocedal and Wright, 2006, Example 17.4). The algorithm iteratively minimizes the AL function with respect to X , using the stationary condition, which yields an update step similar to Equation 4.

While the contribution of regular factors remains unchanged (as described in Equation 5), the contribution of equality factor n at iteration i can be described as shown in Equation 12.

$${\text{H}}_{n}^{i}=||J_{h_{n}}\left(X_{n}^{i}\right)|{|}_{{\text{P}}_{h_{n}}}^{2}$$ (12a)

$$b_{n}^{i}=-{J_{h_{n}}\left(X_{n}^{i}\right)}^{\text{T}}\left({\text{P}}_{h_{n}}h_{n}\left(X_{n}^{i}\right)-\gamma_{h_{n}}\right).$$ (12b)

The AL algorithm implemented in the ecg2o library iteratively solves the inner-loop unconstrained optimization while incorporating both cost and equality factor contributions when constructing the linear system. Upon satisfying the termination criteria or reaching the maximum number of inner-loop iterations, the algorithm exits the inner loop and updates the Lagrange multipliers using Equation 13.

$$\gamma_{h_{n}}^{i+1}=\gamma_{h_{n}}^{i}+{\text{P}}_{h_{n}}h_{n}\left(X_{n}^{i}\right).$$ (13)

Optionally, we increase the penalties according to Equation 14.

$$\rho_{.}=\max\left(\rho_{\max},\alpha \rho_{.}\right),$$ (14)

where $\rho_{\max}$ is the upper bound on the penalty parameter, and α≥1 is the penalty update factor.

### 4.2 KKT-based gauss-newton method

In this section, we present an SQP-inspired method that enables factor graphs to natively solve equality-constrained optimization problems by leveraging KKT conditions. Unlike traditional approaches that require specialized algorithms like Penalty or Augmented Lagrangian methods, our approach demonstrates that factor graphs inherently possess the mathematical structure to handle equality constraints through appropriate factor design, eliminating the need for algorithmic modifications or nested loops.

Our core contribution is the formulation of equality constraints as regular factor nodes that, when combined with standard factor graph optimization, naturally enforce the KKT conditions of the original constrained problem. This transforms the equality constrained problem into an unconstrained optimization over an extended variable space that includes both original variables and Lagrange multipliers. Crucially, this formulation maintains the computational efficiency and sparsity exploitation of standard factor graph optimization while ensuring exact constraint satisfaction—unlike penalty methods that only achieve approximate satisfaction.

The mathematical foundation establishes that standard Gauss-Newton updates applied to our specially designed equality constraint factors yield iterations equivalent to SQP methods. This equivalence represents a significant theoretical advancement, demonstrating that factor graphs can directly implement sophisticated equality-constrained optimization without external algorithmic frameworks.

Consistent with the SQP framework, each subproblem is constructed by linearizing the nonlinear system. We begin by linearizing both the error functions $e_{j}$ and the equality constraints $h_{j}$ around the current estimate $\tilde{X_{j}}$ . Their linearized versions, denoted by ${\hat{e}}_{j}$ and ${\hat{h}}_{j}$ respectively, are given by:

$$e_{j}\left(\underset{X_{j}}{\underbrace{\tilde{X_{j}}+\Delta X_{j}}}\right)\approx {\hat{e}}_{j}\left(\Delta X_{j}\right)=e_{j}\left(\tilde{X_{j}}\right)+J_{e_{j}}\left(\tilde{X_{j}}\right)\Delta X_{j},$$ (15a)

$$h_{j}\left(\underset{X_{j}}{\underbrace{\tilde{X_{j}}+\Delta X_{j}}}\right)\approx {\hat{h}}_{j}\left(\Delta X_{j}\right)=h_{j}\left(\tilde{X_{j}}\right)+J_{h_{j}}\left(\tilde{X_{j}}\right)\Delta X_{j}.$$ (15b)

The Lagrangian function for the linearized system in Equation 15 is given in Equation 16.

$$\hat{L}\left(\Delta X,\gamma \right)=\overset{r}{\underset{j=1}{\sum }}||{\hat{e}}_{j}\left(\Delta X_{j}\right)|{|}_{\Omega_{j}}^{2}+\overset{l}{\underset{j=1}{\sum }}{\gamma_{h_{n}}}^{\text{T}}{\hat{h}}_{j}\left(\Delta X_{j}\right),$$ (16)

Let ΔX*,γ* be the local optimal points. These must satisfy the KKT conditions (Boyd and Vandenberghe, 2004, §5.3.3) of the linearized optimization problem:

$$\nabla_{\Delta X}\hat{L}\left(\Delta X^{*},\gamma^{*}\right)=0,\;\hat{h}\left(\Delta X^{*}\right)=0,$$ (17)

where ∇ , in Equation 17, denotes the gradient, and $\hat{h}$ is the vector of all equality constraints.

Assuming $\gamma *=\gamma^{i}+\Delta \gamma^{i}$ , the KKT system is formulated as:

$$\underset{\text{KKT matrix}}{\underbrace{\left[\begin{array}{cc} {\text{H}}^{i} & {J_{h}\left(X^{i}\right)}^{\text{T}}\\ J_{h}\left(X^{i}\right) & 0 \end{array}\right]}}\underset{\text{update step}}{\underbrace{\left[\begin{array}{c} \Delta X_{gn}^{i}\\ \Delta \gamma^{i} \end{array}\right]}}=\underset{\text{KKT vector}}{\underbrace{\left[\begin{array}{c} b^{i}-J_{h}^{T}\left(X^{i}\right)\gamma^{i}\\ -h\left(X^{i}\right) \end{array}\right]}}$$ (18)

The introduction of equality constraints modifies the linear system compared to unconstrained optimization, as highlighted in red. The key challenge lies in representing these constraints within the factor graph using appropriate variables and factors. Specifically, how can we define the error function and the weighting matrix associated with the equality constraints to enforce them effectively?

#### 4.2.1 Design of equality constraint factors

We propose defining a regular factor for each equality constraint such that it produces the same update step as in (18). Specifically, for an equality constraint $h_{j}$ , we define a regular factors with the following error function and weighting matrix:

$$e_{h_{j}}=\left[\begin{array}{c} h_{j}\left(X_{j}\right)\\ \gamma_{h_{j}} \end{array}\right],\;\Omega_{h_{j}}=\left[\begin{array}{cc} 0_{d\times d} & {\text{I}}_{d\times d}\\ {\text{I}}_{d\times d} & 0_{d\times d} \end{array}\right],$$ (19)

where $h_{j}$ and $\gamma_{h_{j}}$ have the dimension d , and I denotes the identity matrix. This formulation results in a linear system equivalent to the KKT system of the equality-constrained optimization problem. Consequently, it allows us to enforce equality constraints while maintaining an unconstrained factor graph structure. Furthermore, in this approach, the Lagrange multipliers are treated as variable nodes, naturally integrating them into the optimization process.

For ease of implementation, the ecg2o library provides a dedicated class for equality factors, which automatically incorporates Lagrange multipliers and defines the associated weighting matrix described in Equation 19. Additionally, the class simplifies the Jacobian implementation for equality factors, making the equality implementation as straightforward as for regular factors.

This design ensures an efficient and flexible extension of factor graphs, maintaining the computational advantages of existing optimization techniques while enabling constrained optimization in a natural manner.

## 5 Results and evaluation

In this section, we present two case studies: (i) an optimal control problem, and (ii) a comparison between the AL and penalty methods in GTSAM, and the AL and KKT-based methods in ecg2o.

### 5.1 Case study 1

We solve here an optimization problem that generates a sequence of control force inputs for an autonomous car to track a reference velocity trajectory. Our objective is to compare the performance of the AL method with our proposed approach, which models equality constraints as regular edges by incorporating Lagrange multipliers.

#### 5.1.1 Problem formulation

Inspired by Jia et al. (2023), we formulate the optimal control problem as

$$\text{min }\left({\left|\left|x_{N}-r_{N}\right|\right|}_{\text{P}}^{2}\right.+\overset{N-1}{\underset{k=1}{\sum }}\left(||x_{k}-r_{k}|{|}_{\text{Q}}^{2}+||u_{k}|{|}_{\text{R}}^{2}\right)$$ (20a)

$$\text{s.t.}\;x_{k+1}-\left[x_{k}+\frac{\delta t}{m}\left(u_{k}-F_{\text{resis}}\right)\right]=0,\;k=0,\ldots ,N-1,$$ (20b)

where $x_{k}$ represents the vehicle velocity, $u_{k}$ is the input force applied to the car (measured in Newtons), and $r_{k}$ is the reference velocity. In addition, the subscript k denotes the time instance, while δt represents the sampling time of the controller, which defines the horizon length.

The resistance force $F_{\text{resis}}$ depends on the gravitational force $F_{\text{grav}}$ , the rolling resistance $F_{\text{roll}}$ , and the aerodynamic resistance force $F_{\text{air}}$ . The total resistance force is given by

$$F_{\mathrm{resist}}=\underset{F\text{grav}}{\underbrace{m_{v}g\sin\theta }}+\underset{F\text{air}}{\underbrace{\frac{1}{2}\rho_{a}A_{f}c_{a}x^{2}}}+\underset{F\text{roll}}{\underbrace{m_{v}gc_{r}\text{cos}\theta }}.$$ (21)

The model is nonlinear due to the aerodynamic resistance force. The model parameters are as follows: m represents the effective mass of the mass under the effect of the rotational mass of the powertrain, while $m_{v}$ denotes the actual mass of the vehicle. The gravitational constant is given by g , and θ represents the road slope. The air density is denoted by $\rho_{a}$ , and $A_{f}$ is the frontal area of the vehicle. The coefficients $c_{a}$ and $c_{r}$ correspond to the air drag and rolling resistance coefficient, respectively.

**FIGURE 1:**
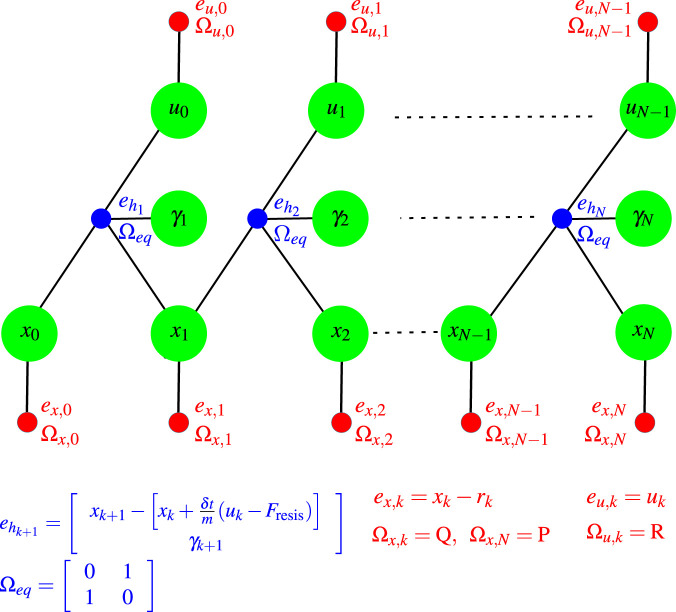
The factor graph for the optimal control problem. The red circles represent the cost terms as regular factors, while the blue circles illustrate the representation of equality constraints as regular factors. k∈ {0, … , N-1}.

The factor graph representing the optimal control problem in Equation 20 is illustrated in Figure 1, where the equality constraints are incorporated using our proposed approach.

#### 5.1.2 Evaluation

**FIGURE 2:**
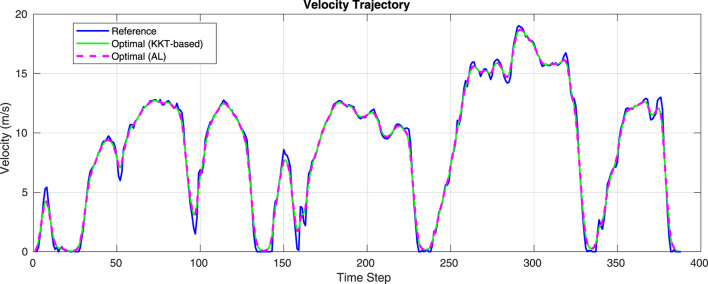
Optimal velocity trajectory obtained from the factor graph-based optimal control problem.

The reference trajectory for the velocity profile consists of 385 points with a sampling rate of 1 s. Using the factor graph-based optimization method, we solve the optimal control problem with weighting parameters Q=P=1000 and R=0.0007 to determine the optimal input sequence. Applying this control sequence results in the optimal velocity profile. The velocity profile corresponding to the optimal control sequence obtained from solving the optimization problem is illustrated in Figure 2. While different controller performances can be achieved with varying parameter settings, our primary focus is to evaluate the optimizer itself. The solution of the optimization problem using the AL method results in a velocity trajectory that is quite similar to our proposed approach. This is because both methods share the same stopping criteria. We computed the root mean squared error (RMSE) between both trajectories to quantify their similarity. The RMSE between the velocity trajectories obtained from the AL method and our proposed approach is 0.2426, indicating a high degree of similarity.

##### 5.1.2.1 Iteration numbers

**FIGURE 3:**
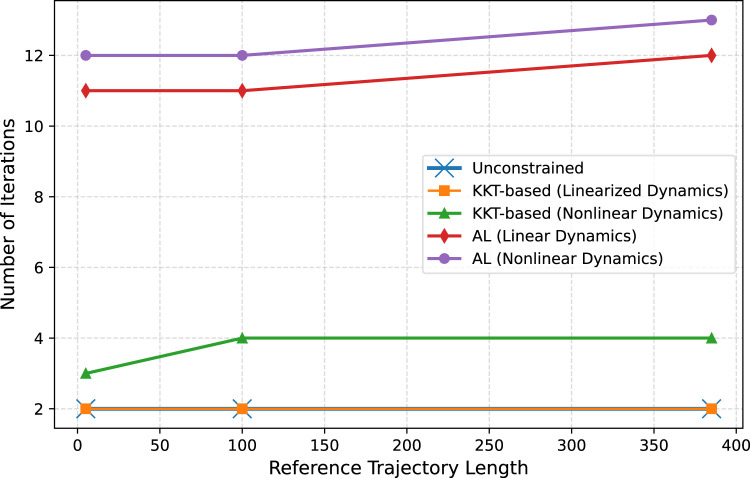
Iteration number across different optimization scenarios and trajectory lengths.

In our case, the presence of nonlinear terms in the equality constraints might influence the number of iterations required for convergence. To address this effect, we considered an approximation for the nonlinear aerodynamic resistance term using a linear model:

$$x^{2}=p_{1}+p_{2}x,$$ (22)

where $p_{1}$ and $p_{2}$ , in Equation 22, are constants. Therefore, we considered five distinct scenarios for comparison. First, we solve the unconstrained optimization problem, where the constraints are ignored, serving as a baseline to understand the effect of enforcing constraints. Next, we apply our proposed method to both a linearized dynamic model and the nonlinear system, allowing us to analyze how constraint approximation influences performance. Additionally, we compare these results with the AL approach, solving the problem in both linearized and nonlinear forms. In addition, we consider three different reference trajectory lengths—5, 100, and 385—to evaluate the optimization performance across varying problem sizes. The results of the iteration number across all scenarios for the three trajectory lengths are summarized in Figure 3.

Figure 3 shows that the iteration number in the unconstrained optimization matches that of our proposed method with linearized dynamics, converging in just two iterations across all trajectory lengths. For nonlinear dynamics, our method requires slightly more iterations (3–4), reflecting the added complexity while maintaining efficiency.

In contrast, the AL method requires 11–13 iterations, approximately three times more than our proposed method, highlighting its higher computational cost. These results were obtained after careful parameter tuning, where we set the maximum number of inner iterations to 1, with $\rho_{\text{init}}=10$ , $\rho_{\text{max}}=50000$ , and α=10 . However, further tuning is still required for different optimization problems.

Regarding sensitivity to the initial value of the Lagrange multiplier, we found that both algorithms maintained a stable iteration number across different Lagrange multiplier initial values.

##### 5.1.2.2 Computation time

**FIGURE 4:**
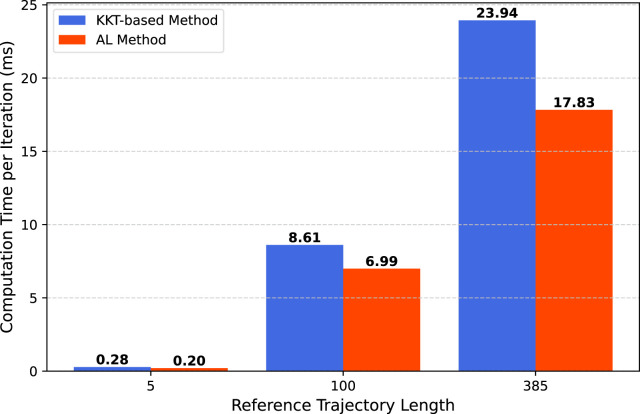
Computation time per iteration for different trajectory lengths using the Proposed Method and Augmented Lagrangian (AL) Method.

We compare the computation time for the nonlinear dynamics scenario across three different trajectory lengths. The optimization problem is solved 1,000 times, and the reported computation time represents the average over these runs to ensure consistency. All experiments were conducted on a Linux system with an Intel Core i9 12th Gen processor and 32 GB of RAM. The results are shown in Figure 4.

The computation time for the AL method is slightly lower than that of our proposed method, which was expected since our solver handles a larger linear system in each iteration. However, advancements in linear solvers have reduced the impact of solving larger systems on overall computation time, making the difference less apparent. The increase in computation time for our method ranges between 20% and 35% , but the significant reduction in the number of iterations makes it a worthwhile trade-off, especially for applications requiring fast convergence.

### 5.2 Case study 2: nonlinear least–squares with an equality constraint

For further validation of the implemented solvers, we adopted a nonlinear equality-constrained problem from the GTSAM library. In this case study, four solvers are considered: Penalty (GTSAM), AL (GTSAM), AL (ecg2o), and the KKT-based method (ecg2o). The constrained optimization problem is formulated in Equation 23.

$$\underset{x_{1},x_{2}}{\min}\;\frac{1}{2}{\left(x_{1}-e^{-x_{2}}\right)}^{2}+\frac{1}{2}{\left(x_{1}^{2}+2x_{2}+1\right)}^{2},$$ (23a)

$$\text{s.t.}\;x_{1}+x_{1}^{3}+x_{2}+x_{2}^{2}=0.$$ (23b)

A key distinction between our AL implementation in ecg2o and GTSAM’s approach lies in the inner iteration control strategy. In the AL method, each outer iteration solves an equivalent unconstrained optimization problem with fixed penalty parameters and Lagrange multipliers. While one could solve this inner problem to high accuracy, this is computationally inefficient since the penalty and multiplier estimates are updated in subsequent outer iterations. Therefore, we explicitly limit the maximum number of inner iterations to balance computational efficiency with sufficient progress toward constraint satisfaction. In ecg2o, we set this limit to five inner iterations per outer loop, whereas GTSAM employs a different convergence strategy without such explicit limits.

**FIGURE 5:**
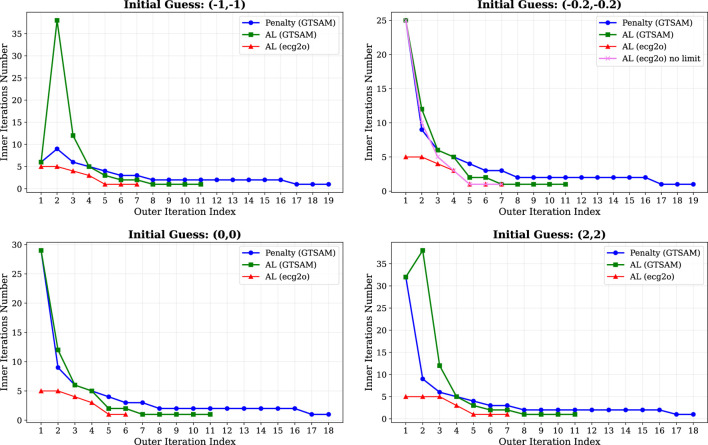
Inner iterations number per outer iteration index for different methods and initial guesses.

This difference is illustrated in Figure 5, which shows the number of inner iterations per outer iteration for each method. The choice of inner iteration limit represents a trade-off: too few iterations may hinder progress, while too many may waste computational effort on prematurely accurate inner solutions. Notably, when we increased the maximum inner iteration limit from 5 to 50 in our AL implementation for the initial guess (−0.2,−0.2) , the total iteration count increased from 20 to 45 without additional improvement in solution quality.

The iteration numbers reported in Table 1 are measured consistently across implementations. In ecg2o, we directly count the number of inner iterations (i.e., iterations where the main calculations and linear system updates occur), while for GTSAM we sum the reported *uopt_iters* across all outer iterations to obtain a comparable metric. All solvers successfully converged to the optimal solution x*=(0,0) with accuracy better than $10^{-4}$ across all initial guesses

$$x^{\left(0\right)}\in \left\{\left(-1,-1\right),\;\left(-0.2,-0.2\right),\;\left(0,0\right),\;\left(2,2\right)\right\}.$$

The KKT-based Gauss-Newton method in ecg2o achieved the fastest convergence (average of five iterations), followed by AL in ecg2o (average of 20 iterations). Both GTSAM baselines required substantially more iterations (averages of 73 for penalty and 72 for AL). The performance advantage of our KKT-based approach is consistent across initial conditions, demonstrating its efficiency for this class of constrained problems.

**TABLE 1:** Case study 2: iterations number to convergence for each method and initial guess.

Method	$x^{\left(0\right)}=\left(-1,-1\right)$	$x^{\left(0\right)}=\left(-0.2,-0.2\right)$	$x^{\left(0\right)}=\left(0,0\right)$	$x^{\left(0\right)}=\left(2,2\right)$	Avg.
Penalty (GTSAM)	56	75	79	82	73.0
AL (GTSAM)	72	57	61	98	72.0
AL (ecg2o)	20	20	19	21	20.0
KKT-based (ecg2o)	6	4	1	9	5.0

We further investigated the effect of the penalty parameter α on AL performance. Increasing α from 1.5 to 2 reduced the iteration count from 20 to 18 for the (−0.2,−0.2) case, as accelerated penalty growth drives faster constraint satisfaction. However, overly aggressive penalty increases risk numerical ill-conditioning, particularly in large-scale systems. This parameter sensitivity highlights a key advantage of our KKT-based method, which requires no such tuning while maintaining robust performance. The AL parameters used in this study were $\rho_{\text{init}}=1$ , $\rho_{\text{max}}=50000$ , and α=1.5 , with a maximum of five inner iterations per outer loop.

## 6 Conclusion and future work

In this paper, we addressed the challenge of incorporating equality constraints into factor graphs, an essential extension for applications beyond robotic perception, such as optimal control. While the AL method is the state-of-the-art approach for handling equality constraints, we proposed an SQP-inspired, KKT-based method that natively integrates constraints within factor graph optimization without requiring additional iterative adjustments.

The proposed approach outperforms the AL method by achieving faster convergence and eliminating the need for parameter tuning, thereby enhancing its practicality. Its efficiency and robustness were validated through a case study on velocity tracking in autonomous vehicles. In addition, the implementation was compared against the Penalty method and AL in GTSAM, demonstrating superior performance.

As future work, we aim to explore more advanced constrained optimization techniques beyond AL, specifically targeting the handling of inequality constraints within factor graph frameworks. This would further expand the applicability of factor graphs to control and planning problems in robotics.

## Data Availability

The original contributions presented in the study are included in the article/supplementary material, further inquiries can be directed to the corresponding author.
